# 3D Bioprinting Strategies for the Regeneration of Functional Tubular Tissues and Organs

**DOI:** 10.3390/bioengineering7020032

**Published:** 2020-03-31

**Authors:** Hun-Jin Jeong, Hyoryung Nam, Jinah Jang, Seung-Jae Lee

**Affiliations:** 1 Department of Mechanical Engineering, Wonkwang University, 460, Iksan-daero, Iksan-si, Jeollabuk-do 54538, Korea; hunjinjeong312@gmail.com; 2 Department of Creative IT Engineering, Pohang University of Science and Technology, 77 Cheongam-Ro, Nam-Gu, Pohang, Gyeongbuk 37673, Korea; alfzmtea@postech.ac.kr; 3 School of Interdisciplinary Bioscience and Bioengineering, Pohang University of Science and Technology, 77 Cheongam-Ro, Nam-Gu, Pohang, Gyeongbuk 37673, Korea; 4 Department of Mechanical Engineering, Pohang University of Science and Technology, 77 Cheongam-Ro, Nam-Gu, Pohang, Gyeongbuk 37673, Korea; 5 Institute of Convergence Science, Yonsei University, 50, Yonsei-ro, Seodaemun-gu, Seoul 03722, Korea; 6 Department of Mechanical and Design Engineering, Wonkwang University, 460, Iksan-daero, Iksan-si, Jeollabuk-do 54538, Korea

**Keywords:** 3D bioprinting, biocomposite ink, tubular tissue, tubular organ

## Abstract

It is difficult to fabricate tubular-shaped tissues and organs (e.g., trachea, blood vessel, and esophagus tissue) with traditional biofabrication techniques (e.g., electrospinning, cell-sheet engineering, and mold-casting) because these have complicated multiple processes. In addition, the tubular-shaped tissues and organs have their own design with target-specific mechanical and biological properties. Therefore, the customized geometrical and physiological environment is required as one of the most critical factors for functional tissue regeneration. 3D bioprinting technology has been receiving attention for the fabrication of patient-tailored and complex-shaped free-form architecture with high reproducibility and versatility. Printable biocomposite inks that can facilitate to build tissue constructs with polymeric frameworks and biochemical microenvironmental cues are also being actively developed for the reconstruction of functional tissue. In this review, we delineated the state-of-the-art of 3D bioprinting techniques specifically for tubular tissue and organ regeneration. In addition, this review described biocomposite inks, such as natural and synthetic polymers. Several described engineering approaches using 3D bioprinting techniques and biocomposite inks may offer beneficial characteristics for the physiological mimicry of human tubular tissues and organs.

## 1. Introduction

Tubular tissues and organs exist with various forms and functions in the gastrointestinal (esophagus, intestines), respiratory (trachea), vascular (veins, arteries), and urinary (bladder, urethra) systems [1]. These tubular tissues have various diseases and malfunctions requiring appropriate therapeutic interventions, such as donor tissue transplantation, autologous implant, and replacement with a synthetic prosthesis. Autologous transplantation is considered as one of the best therapeutic methods; however, in the case of the trachea and esophagus tissue with little redundancy and non-existent autologous tissues, therapeutic approaches using donor tissues or synthetic prosthesis are required [2,3,4]. Donor tissue transplantation is an ideal option, but there remains a disparity between the number of the appropriate donors and the high demand for the therapeutic use of donor tissue [5,6,7]. In addition, finding a suitable donor tissue is not easy since most of the tubular tissues are associated with poor prognosis after surgery [4,8]. For these reasons, many tissue engineering approaches have been researched for the manufacture of suitable tubular tissues and organs.

Successful bladder tissue engineering using tissue-engineered hollow spherical biodegradable structures was first reported in 1996 [9]. Since then, many studies have been reported to create artificial functional tubular tissue, such as tracheal tissues. However, these tubular tissues generally have similar morphological features, but the fabrication of tubular tissues requires high-level microfabrication-techniques due to their complex hierarchical macro- and micro-structure containing the different cell types and extracellular matrix (ECM) [10,11,12,13].

Various microfabrication techniques for tissue engineering, such as electrospinning [14,15,16,17,18,19], cell-sheet engineering [20,21,22], and mold-casting [23,24,25,26], have been widely studied to make complex multi-layered architecture for artificial functional tubular tissues. These approaches would use additional substrates (e.g., rotating rod, sacrificial mold) to create the tubular architecture, as well as requiring complex and multiple manufacturing processes. Besides, these approaches are not only insufficient to create the tubular structure with a target-specific mechanical property but also restrict shape-freedom due to the technical limitations.

3D bioprinting technique is emerging as an alternative to overcome the limitations of fabrication in terms of building tubular tissues and organs [27,28]. 3D bioprinting has also been utilized for higher-complexity structures with printable biocomposite inks containing the living cells and natural and synthetic polymers [29]. The greatest benefit of 3D bioprinting technology in tubular tissue engineering is the ability to fabricate tubular structures with multi-layer free-form constructs, as well as allowing the placement of biomaterials in a cell and printable ink containing the biochemical microenvironmental cues [10]. In particular, this technology has unlimited possibilities that are feasible for producing complex tissues and organs. Additionally, it can be applied anatomically and clinically since it should facilitate the manufacture of patient-tailored 3D structures [30,31,32].

Therefore, this review dealt with the state-of-the-art of 3D bioprinting technologies, various biocomposite inks, and their applications to tubular tissue engineering focused on a blood vessel, trachea, and esophagus tissue regeneration.

## 2. 3D Bioprinting Techniques 

To achieve the building of 3D-engineered human tissue and organ analogs, it is necessary to use accurate and well-controlled fabrication methods for suitable biomaterials and living cells. Due to these functional 3D fabrication requirements, four types of 3D bioprinting techniques have been developed based on the principle of releasing the printable biomaterials from the printing head.

### 2.1. Extrusion-Based Bioprinting Systems

**Figure 1:**
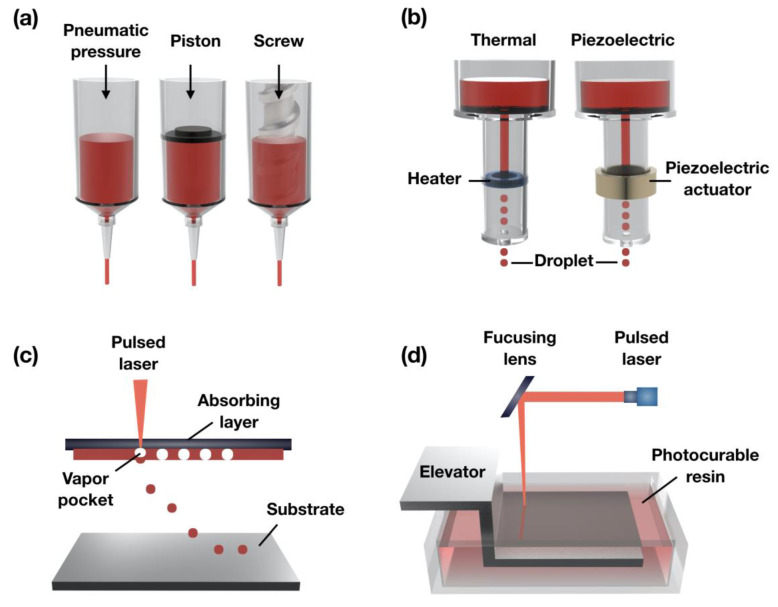
Schematic illustration of the (**a**) extrusion-based; (**b**) ink-jet; (**c**) assistant laser; and (**d**) stereolithography-based 3D bioprinting systems.

The principles of extrusion-based bioprinting are dispensing biomaterials through the nozzle by physical force (e.g., pneumatic pressure, piston, or metal screw) and pneumatic pressure (Figure 1a). The extrusion head moves in the x, y, and z directions under the instruction of the CAD-CAM software to produce a 3D architecture by staked biomaterial onto the substrate. Even if this technique has a lower accuracy than the other 3D bioprinting methods (ink-jet, laser-based), it can be capable of various biomaterials, such as cell-laden bioink, cell-spheroids, hydrogels, and high-viscosity thermoplastic polymers. This bioprinting method allows the extrusion of an extensive range of viscous materials (6–30 × 10^7^ mPa·s), and the resolution of the extruding in the exit of the nozzle is in the range of 100 µm–millimeter [33,34]. Among them, thermoplastic polymer, such as polycaprolactone (PCL) [35,36,37], poly (lactide-co-glycolic-acid, PLGA) [38,39], poly (L-lactic acid, PLLA) [40,41], have been widely applied to fabricate hard-tissue and sturdy supporting constructs. The distinct advantage of this bioprinting technique is that it can be installed in a multi-head system, allowing the simultaneous use of one or more biomaterials, such as synthetic polymer and cell-laden bioink. Therefore, given the ability to quickly manufacturing complex 3D tissue structures that morphologically and biologically mimic the human body, the extrusion technique is regarded as a promising clinical approach [30].

### 2.2. Ink-Jet Bioprinting Systems

Ink-jet bioprinting makes use of mechanical pulses, such as piezoelectric and thermal, to manufacture small-sized bioink droplets (spot size resolution of around 50–75 µm) and is referred to as the drop-on-demand method (Figure 1b). In the case of the piezoelectric ink-jet, break piezoelectrical materials are made by generating acoustic waves using an actuator to create droplets [42]. The thermal ink-jet uses the generated small air bubbles by applying electrical heat within a heated printhead to make droplets. These air bubbles are able to control the material being extruded from the exit of the print nozzle [43]. Cell viability in ink-jet bioprinting may differ depending on the applied mechanical pulses.

The main advantage of the ink-jet technique is that it enables to print with picoliter-volume droplets to build micro-structures because it can control the desired ejected droplet size as a variation of ultrasound parameters, such as amplitude, pulse, and time. However, there are drawbacks, including the need for low-viscosity material (3.5–12 mPa·s) to avoid clogging, and bioprinting material should be quickly gelated in post-print for 3D build constructs. In addition, the mechanical property of the post-printed construct has weak solidity, and the drying of a printed droplet on the substrate during bioprinting is a problem to be solved.

### 2.3. Laser-Assisted Bioprinting

Laser-assisted bioprinting uses a laser source to fabricate at high precision onto substrates. There are two separate approaches: laser-guided direct writing and laser-induced forward transfer (LIFT) [44,45]. Compared to other printing approaches, laser-assisted printing was rarely used in the old days; however, it has recently become increasingly popular as the 3D printing method for microfabrication.

This technique consists of a focusing system (to align and focus laser), an absorbing layer (ribbon), a pulsed laser beam (to induce the transfer of bioink), and a substrate for the bioink layer (Figure 1c) [46]. In brief, the laser pulse is induced on the absorbing layer to create a high-pressure bubble from the bioink layer and then drop the bioink onto the substrate. This bioprinting method can also use bioink with the high-viscosity and high-concentration of the cell (1–300 mPa·s) since it is a nozzle-free system [47]. Therefore, the formation of delicate shaping and arrangement of the cell patterning can be achieved while bioprinting without affecting cell viability. However, the fast gelation of bioink is essential for achieving a highly precise shape. For this reason, the laser-assisted bioprinting is a time-consuming process because of the relatively low flow rate for the crosslinking of the material. Therefore, the challenge of building 3D constructs of clinically relevant size remains [48].

### 2.4. Stereolithography-Based Bioprinting

Stereolithography (SLS or SL) is a form of 3D bioprinting technology using a laser source (ultraviolet, infrared radiation) for building 3D structures. An SLS bioprinting system consists of the light source, a reservoir with the liquid photocurable resin, an elevator system, and a digital mirror device (DMD) [49]. This technique was developed commercially in 1986 by Chuck Hill and then widely used for creating prototypes and complex production parts (Figure 1d). It can allow relatively rapid manufacturing time since the whole layers of the 2D slicing pattern of the 3D constructs are irradiated in a photopolymer reservoir. Also, this system can fabricate the sub-micron structure with highly precise 3D shapes (~1.2 µm) because a laser source can be focused on a small spot in the photocurable resin [50].

As this stereolithography bioprinting technology has been applied in the field of tissue engineering, various materials have been developed to contain bioink and cells with photo-initiators. In particular, commonly used photocurable materials in stereolithography bioprinting technique include the acrylics and epoxies. However, to apply the tissue-engineered approaches, the biocompatible photocurable resin needs to contain propylene fumarate (PPF) and trimethylene carbonate (TMC). These biocompatible photocurable materials have been widely used to manufacture with complex architecture and sacrificial mold [51]. However, SLS 3D bioprinting is still challenging due to cytotoxicity of the photocurable resins and high cost for system installation.

## 3. Printable Biocomposite Inks for Various 3D Bioprinting Techniques

Printable biocomposite inks are generally classified as natural, synthetic, and functional polymers. Natural polymers (e.g., collagen, gelatin, alginate, ECM-based ink) have been widely used in the field of the tissue-engineering and have been considered promising biomaterials with similar components of native tissue or organs in the human body [52,53,54]. In particular, protein-based natural polymers, such as collagen, gelatin, and ECM-based ink, have a remarkable capacity to help regenerate the epithelial layer, which is essential for creating the functional tubular tissue. Alginate bioink has an inferior biological activity compared to protein-based natural polymers. However, it has been widely utilized as bioprinting material to build the tubular constructs due to the easily controllable printability and excellent biocompatibility.

Ideally, synthetic polymers support the structure of the 3D printed target-tissue and degrade completely after implantation without side effects. Also, synthetic polymers have to pass the verification of strict criteria to be applied in clinical settings. In this section, we have described representative biocompatible synthetic polymers, such as thermoplastic polycaprolactone (PCL), polyethylene glycol (PEG), and polylactic acid (PLA), which have been recognized by the Food and Drug Administration (FDA) or widely applied in the field of tubular tissue-engineering [55,56,57,58]. In addition, we previously reported the combinations of natural polymer and synthetic polymer or nanocellulose that have been tried for enhancing mechanical properties and positive biochemical factors [59].

### 3.1. Natural Polymers

#### 3.1.1. Collagen

Collagen is a representative natural polymer applied to bioprinting, and it consists of proline and glycine, with a triple-helix arrangement of polypeptides, as the most abundant protein in the human body [52,60]. In human organs (e.g., skin, bone, cartilage, vessel) and connective tissues, various collagen types exist, such as collagen I, III, and IV [61]. Among them, collagen type I is the most abundant and also the most commonly used in 3D bioprinting [62]. Collagen has characteristics of little cross-species immunological reaction and low toxicity, as well as allowing enhanced cell attachment and proliferation due to the presence of asparagine-glycine-aspartic acid (RGD) residues [11]. For this reason, collagen-based bioinks are regarded as highly promising biomimetic materials.

The main advantage of the collagen-based bioink is that it enables the embedding of living cells with ECM components and biochemical materials. However, because it has a crosslinking property, the use of a crosslinker or gelation process by temperature is essential for the construction of the 3D structure. In addition, the mechanical strength and bioprinting property of the collagen-based bioinks are dependent on viscosity due to collagen contents. For this control, many studies have combined collagen with biocomposite materials, such as fibrin [63,64], alginate [65], chitosan [60], and agarose [66], for improving printability and mechanical properties. Collagen-based bioink is undoubtedly an excellent biomaterial, but there remains scope for the improvement for use as bioprinting materials.

#### 3.1.2. Gelatin

Gelatin is a type of protein obtained from collagen as a partially hydrolyzed form, and it is a biodegradable and biocompatible natural polymer [67]. Likewise, as with collagen, gelatin-based bioink can enhance cell attachment and proliferation because it has an RGD sequence with abundant integrin-binding motifs. Gelatin is dissolved in water to maintain the thermo-sensitivity property, but it reversibly forms a low-viscosity soluble state at human body temperature [68]. Because of these limitations in maintaining the form, using only the gelatin-based bioink as a printable biomaterial is not suitable to build sturdy 3D tissue structures. Therefore, many studies have attempted the development of the printable gelatin-based composite ink mixed with other polymer materials, such as PCL [69,70], chitosan hydrogel [71], hyaluronic acid [72], fibrin [73], alginate [74,75], and silk [76,77], for improving structure’s stability.

Gelatin methacrylate (GelMA) has been widely used as an advanced bioink that modifies photocrosslinkable polymers [78]. The mixture of GelMA with photoinitiator (e.g., 2-hydroxy-1-(4-(hydroxyethoxy) phenyl)-2-methyl-1-propanone (Irgacure 2959)) undergoes rapid crosslinking after extrusion through exposure to UV light (360–480 nm in wavelength). This crosslinkable property enables the structural stabilization after bioprinting. Also, GelMA has excellent biological characteristics of cell adhesion, biodegradability, and cell migration because it involves collagen and gelatin components, such as integrin-binding motifs and RGD. Due to these promising properties, many studies have applied the combined material for improving the desired quality.

#### 3.1.3. dECM Ink

dECM-based ink has been regarded as a promising material for 3D bioprinting [79]. dECM-based ink is fabricated by the decellularization process of the target-tissue. It has an inherent component of tissue-specific microenvironment cues, such as proteoglycans, glycoprotein, and collagenous protein. To date, various dECM-based inks have been reported for target-specific tissues, such as derived skin [80], bone [39], vessel [59], liver [81], kidney [82], and so on. Each derived tissue has different printability properties, but all have the distinguishing feature of temperature-responsive gelation under the physiological environment [83].

#### 3.1.4. Alginate

Alginic acid, also called alginate, is an anionic polysaccharide distributed in the cell walls of brown algae [84]. It has a hydrophilic property and forms a viscous gel when hydrated. Alginate hydrogel has been applied as a wound dressing material because it has good biocompatibility and is structurally similar to natural ECM with a bioinert property [85]. In addition, alginate has been extensively used as an ink to fabricate the 3D structure in the field of tissue engineering because it can robustly form a cell-compatible hydrogel by instantly polymerizing using multivalent cations (e.g., Ca^2+^, Ba^2+^). Given their facilitation of tissue formation, hydrogel inks have been modified for a variety of tissue-engineered approaches, such as bone [86], cartilage [87], and vascular tissue [88]. In addition, because the alginate has no cell-adhesive site, the bioactive component enables the addition of the signal trigger, such as RGD, for cell viability and differentiation [89].

### 3.2. Synthetic Polymer

#### 3.2.1. Polycaprolactone

PCL is one of the aliphatic polyesters; it is the most frequently used biomaterial for 3D bioprinting in the field of tissue engineering. PCL has superior printability due to its low melting temperature and glass-transition temperature. In addition, it is well known as a clinically applicable biomaterial approved by the FDA as a biocompatible and biodegradable polymer [90].

The degradation rate of biomaterials must be carefully considered before the fabrication of the target-specific tissue-engineered structure. If using the 3D scaffold with quickly degradation materials, there is a possibility of the mechanical property rapidly degrading after implantation in the body. In this regard, PCL has a great benefit as it can control the degradation rate by blending of different ratios of the polymer and copolymers [91,92]. The degradation mechanism of the PCL has a bulk erosion process by hydrolysis, and, in this process, PCL does not release toxic components [93,94]. Because of these convenient advantages, PCL is actively utilized as various bioprinting materials.

#### 3.2.2. Polylactic Acid 

Polylactide or polylactic acid (PLA) is most widely used for creating tissue-engineered architecture [95]. Also, PLA has been approved by the USA FDA for human clinical applications. The PLA has been used as a biomaterial for frequency 3D bioprinting because of its readily available thermoplastic properties [96]. Although there are differences depending on molecular weight (MW), PLA has relatively high mechanical properties, with an approximate tensile modulus of 3 GPa and tensile strength of 50–70 MPa [97]. The MW has a significant effect on biodegradability, but high-MW PLA is likely to cause inflammation and infection in vivo [98]. Therefore, before 3D bioprinting, the MW property must be considered for the mechanical properties of the target tissue.

#### 3.2.3. Polyglycolic Acid 

Polyglycolic acid (PGA) is a thermoplastic material with a high melting point and glass transition temperature, and it is more acidic and hydrophilic than PLA [99]. In addition, it is used as a surgical suture fiber because of its high mechanical strength and biocompatibility [100]. In the field of tissue engineering, solvent casting and compression molding are used to create PGA-based porous scaffolds [56]. However, PGA requires precise control as it is highly sensitive to degradation. Additionally, glycolic acid produced during the biodegradation process can be absorbed into the body, but the increased acid concentrations in the surrounding tissues may cause tissue damage.

### 3.3. Functional Polymer

As mentioned above, generally, biocomposite inks are classified as natural- and synthetic-based polymers, and these have been attempted to be used for complex and cell-compatible 3D constructs as tissue-engineering approaches [101]. Among them, hydrogel-type inks (i.e., alginate, collagen, dECM ink) have been considered as attractive materials because these can provide an optimized environment to a living cell. However, to be suitable for 3D bioprinting, these hydrate materials require adequate rheological properties to keep shape during bioprinting and must have cross-linking abilities, allowing to retain the 3D structure fidelity after bioprinting. Recently, the importance of versatile bioink materials in the field of tissue engineering has led to the development of functional polymers with improved biocompatibility, rheological behavior, and mechanical properties [102]. In this section, functional polymers that improve the bioprinting stability and fidelity when combined with nanocellulose biomaterials are introduced.

Nanocellulose refers to cellulosic nanomaterials, including cellulose nanocrystals (CNC) and cellulose nanofibrils (CNF) [103,104,105]. Gary Chinga-Carrasco et al. developed printable ink with bagasse, which is an underutilized agro-industrial residue [106]. This functional polymer has demonstrated non-cytotoxicity, stable bioprinting property, and shape fidelity, as well as potential that a low-value agro-industrial residue (bagasse) can be converted into a high-value product as disposable bioinks for 3D bioprinting. However, further evaluation is required for clinical applications. Kajsa Markstedt et al. developed functional bioink that combines the outstanding shear thinning properties of nanofibrillated cellulose (NFC) with the alginate [105]. The nanocellulose-based bioink enables fidelity bioprinting of 2D structure as well as 3D construct, which is anatomically the shape of a human ear and sheep meniscus. Also, nanocellulose-based bioink exhibits excellent cell viability. Therefore, these functional polymers using cellulose nanofibrils have shown promising potential as 3D bioprinting materials.

## 4. Recent Design Approaches for Engineering Tubular Structures

Tubular tissues and organs, such as the gastrointestinal tract, urinary tract, and respiratory tract, exist everywhere in the human body, serving major functions, including distributing fluids and air through the organs [107]. Almost all of the tubular tissues have a multilayer cellular structure from the innermost to the outermost, and the inner structure has an endothelium cell layer [6]. There are no existent pioneering fabrication techniques to fabricate tubular-shaped tissues and organs in the field of tissue engineering [5]. However, various fabrication methodologies have been suggested for constructing tubular structures with mimicking the inherent multilayer cellular constructs.

**Figure 2:**
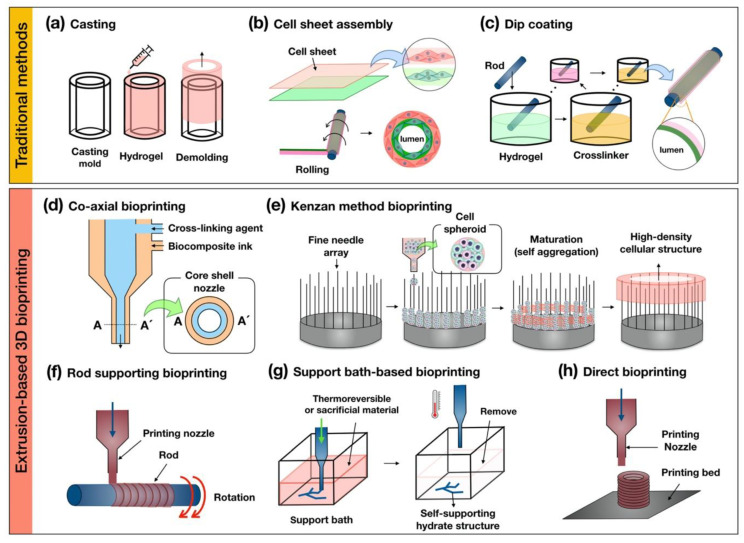
Schematic illustrations of the traditional methods of (**a**) casting; (**b**) cell sheet assembly; (**c**) dip coating and the extrusion-based 3D (**d**) co-axial; (**e**) kenzan method; (**f**) rod supporting; (**g**) support bath-based; and (**h**) direct bioprinting for fabricating tubular structures.

Traditional methods, such as casting, cell sheet assembly, and dip coating, have been attempted to create the tubular structures. The casting method creates the tubular structure by the biomaterials filling in the sacrificial mold and then demolding after appropriate chemical processes, such as gelation or crosslinking (Figure 2a). This method was proposed in 1986 by Weinberg and Bell, who made artificial vascular structures using the collagen-containing fibroblast and smooth muscle cells [23]. Since then, cell sheet assembly technology has been reported for reproducing hierarchical multi-layered cellular structures (Figure 2b) [20,21,22]. This method has been facilitated for creating the multilayer tubular structure by rolling on the rod using the stacked monolayer fabricated by biological functional materials containing extracellular matrix and target-specific cell components. The dip-coating method can also produce multiple tubular structures using rods by repeatedly dipping in the hydrogel and cross-linker agent (Figure 2c) [108,109,110]. These traditional methods have shown the promising ability to mimicking the cellular arrangement of native tubular tissues. However, there has been an unmet challenge in implementing physiological and mechanical properties suitable to tissue-specific complex environments. Also, these methods have unavoidable hurdles for fabricating shape-free forms and controllable structures. To overcome these challenges, hybrid-type technology, combining the traditional method with 3D bioprinting, has been tried to fabricate a free-form tubular structure [111]. Hybrid-type approaches have shown the possibility of creating a free-form tubular construct, although the structure has not been directly printed.

3D bioprinting technology has been emerging as a promising approach to facilitate complex structures and spatial cell positioning in tubular tissue engineering [112,113]. Among the various 3D bioprinting techniques (e.g., extrusion-based, ink-jet, laser-assisted, stereolithography-based 3D bioprinting), extrusion-based 3D bioprinting has been one of the most utilized for creating the tubular structure because it is relatively convenient for the installation of the system and availability of a wide range of biomaterials. In this article, we summarized and classified several categories of the extrusion-based 3D bioprinting for building a multilayer tubular structure (co-axial-, kenzan method-, rod supporting-, support bath-, direct bioprinting) (Table 1).

The co-axial bioprinting method is capable of creating a tubular structure using a core–shell nozzle that is capable of extruding two or more biomaterials (Figure 2d). Several research teams have been employing this method to print complex tubular structures with biocomposite inks. Gao et al. developed the printable hybrid bioink containing a mixture of vascular tissue-derived decellularized extracellular matrix (VdECM), alginate, and human umbilical vein endothelial cells [59]. Subsequently, they fabricated a perfusable multilayer blood vessel by co-axial bioprinting [88]. Yongxiang Luo et al. printed a 3D porous scaffold with regular macropores and a network of a controllable hollow structure as an embedded vasculature-like system using co-axial bioprinting [114]. This method can allow not only the building of a hollow construct with functional biological components but is also capable of fabricating permeable vascular-embedded 3D constructs. Moreover, it can manufacture small-diameter vascular structures with endothelial and smooth muscle layers, as well as being able to print long-length warping vascular structures with a minimal amount of time. However, this method has limitations in terms of making the anatomical bifurcate structure and stacking hierarchical constructs.

Kenzan method bioprinting was invented by Koich Nakayama. It can create a high-density cellular structure by locating cell spheroids on a fine needle array (Figure 2e) [115,116,117,118]. The main principle of this method uses the natural and intrinsic feature of cell-to-cell self-aggregation. Recently, this research group fabricated the esophagus-like tubular structure without scaffold using the multicellular spheroids that maturated during several periods in the bioreactor to create the rigid organoids.

The rod supporting bioprinting method produces a hollow construct by dispensing printable biomaterials on a rotating rod (Figure 2f). The rotating rod is provided as temporary support to the printed biomaterial for keeping a 3D shape and is removed when the printed structure is considered to be self-supporting. Sang-Woo Bae et al. printed the artificial tracheal structure with a synthetic polymer (i.e., PCL) and cell-laden bioink (epithelial cells and bone-marrow stem cells) by sequential extruding on the rotating rod [119]. Qing Gao et al. fabricated a hydrogel-based vascular structure with multilevel fluidic channels using a combination with co-axial bioprinting [31]. The main advantage of this bioprinting method is the manufacturing ability of the self-supporting multi-layer hollow structure with target-specific cell components though sequential bioprinting using above two or more printable biomaterials, such as polymer-based and cell-laden bioink. However, there is a disadvantage in that the figuration of the printed structure is dependent on the rotating rod shape.

The support bath-based bioprinting method refers to using a thermo-sensitivity gel bath or sacrificial materials for supporting the bioprinting biomaterials (Figure 2g). This method obtains a self-supporting structure, keeping the reversible condition for removing the biomaterials after bioprinting in the gel bath. Thomas J. Hinton et al. introduced this bioprinting method as the freeform reversible embedding of suspended hydrogel (FRESH), which uses this technique to print the biomimetic section of the human right coronary arterial tree with alginate-based bioink [120]. The research team, Tal Dvir et al. printed the endothelial cell-laden hydrogel to create the blood vessel-embedded cardiac tissue of the rabbit scale in the supporting bath with an aqueous solution containing sodium alginate, xanthan gum, calcium carbonate [121]. This method enables 3D bioprinting of the hydrate biomaterials, including alginate, collagen, and fibrin. Also, it can create complex 3D anatomical architectures, including branched coronary arteries, embedded vascular system organoids, etc. These results have demonstrated the potential of the approach for engineering personalized tissues and the bioprinting of patent-tailored biochemical microenvironment.

Direct bioprinting can be used to stack the printable biomaterials layer-by-layer for building the 3D structure (Figure 2h). In order to use this bioprinting technique, biomaterials must have sufficient solidity properties to maintain the 3D structure. For bioprinting hydrate bioink, in particular, it is essential to consider the rheological properties of materials during the bioprinting process. Anthony Atala et al. used direct bioprinting to build a 3D urethra tubular scaffold with a polymeric framework and cell-laden fibrin hydrogel [122]. The polymeric framework consisting of the PCL and PLCL (polylactic acid-co-ε-caprolactone) stably supports the printed hydrogel bioink for the desirable fabrication of the 3D tubular structure. Recently, Yifei Jin et al. developed self-supporting hydrogel by mixing laponite nanoclay and then successfully printed sturdy architecture without supporting the gel bath and polymeric structures [123].

## 5. Application of the 3D Printed Tubular-Organs with Various Biocomposite Inks 

### 5.1. Esophagus

The esophagus is one of the gastrointestinal tracts and a 20–25 cm hollow structure, connecting the oropharynx and the stomach [124,125,126]. It allows the transport of food to the stomach by peristalsis and contractions of the muscle layer. Because the esophagus has a complex hierarchical structure (mucosa, submucosa, muscle layers), this must be considered when fabricated using engineering approaches [127,128,129].

Every year, 5000 to 10,000 patients are diagnosed with an esophageal disease requiring partial repair or full-thickness circumferential replacement, such as esophageal cancer, malignancy, congenital long-gap atresia, and esophageal achalasia. The strategy of the esophageal treatment is normally a gastric pull-up or autotransplantation using intestine or skin. In the case of autograft, using the gastrointestinal tract is an unavoidable strategy to achieve circumferential full-thickness repair since there is no substitute for esophagus tissue. However, although the autograft might allow the transfer of liquids or solid matter, complete restoration of the native tissue is compromised. Therefore, several approaches using 3D bioprinting technology have been researched to achieve the esophageal substitution, which replicates primary histological features of hierarchical cellular structures (Table 2).

**Figure 3:**
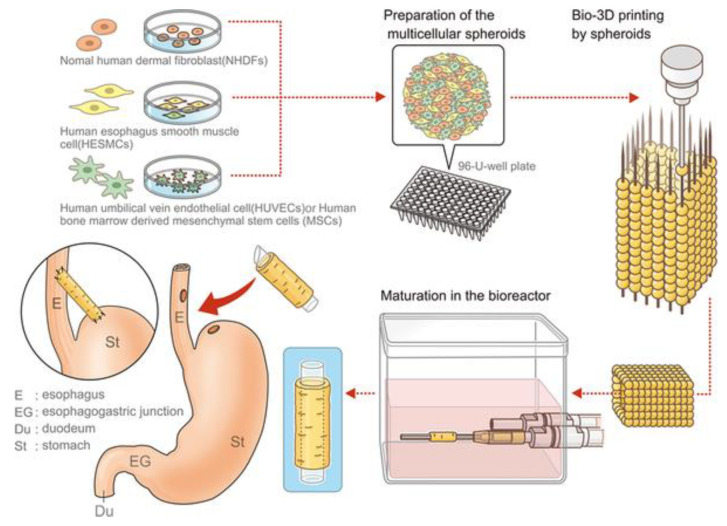
Schematic illustration of the esophageal tubular structure by kenzan method bioprinting [115].

Yosuke Takeoka et al. developed a scaffold-free biomimetic structure for the regeneration of the esophagus using the kenzan method bioprinting (Figure 3) [115]. This team used the maturated cell spheroids of the normal human dermal fibroblasts (NHDFs), human esophageal smooth muscle cells (HESMCs), human bone marrow-derived mesenchymal stem cells (MSCs), and human umbilical vein endothelial cells (HUVECs) to print the tubular multicellular structures. Mechanical and histochemical assessment of the printed esophagus-like tubular structure had been done with the content ratio of those cell sources. The high proportion of mesenchymal stem cell groups tended to give greater mechanical strength as well as the expressed α-smooth muscle actin and vascular endothelial growth factor (VEGF) on immunohistochemistry. After bioprinting of esophagus-like scaffold-free tubular structures with demonstrated multicellular proportion, it was matured in bioreactor and then transplanted into rats as esophageal grafts. The esophageal grafts were implanted between the stomach and esophagus with a silicone tube. Results showed the grafts were maintained in vivo for 30 days, and the epithelium extended and covered the inner lumen and was able to pass food as well. The epithelialization of the inner surface of the esophageal lumen should be considered as the key regenerative factor because it must be done postoperatively with non-sterilized solid matter. In this respect, this research result has been promising as a potential substitute for esophageal transplantation using bioprinting.

In Gul Kim et al. employed both techniques of the supporting rod bioprinting and electrospinning to build the enhanced tubular structure with two-layer [15]. That study evaluated the 3D printed esophageal graft and the effect of bioreactor cultivation on cell maturity for muscle regeneration and epithelialization. To fabricate the tubular framework, the membrane was manufactured by electrospun polyurethane (PU) on the rotating rod (diameter: 2 mm), and then to improve the mechanical stability, the PCL strand was squeezed using the extrusion-based system. Cell-seeded (hMSCs) tubular frameworks were maturated in the customized bioreactor system, and shear stress of the 0.1 dyne/cm^2^ flow-induced with a pattern of 1 min/2 min for engagement/resting was applied to invigorate the frameworks. In comparison results of the histological analysis in the circumferential esophageal defects in a rat model from bioreactor cultivation and the omentum-cultured groups, both the groups showed over 80% mucosal regeneration without a fistula. The follow-up study by this research group suggested the further extended bioreactor culture system that could apply the different mechanical stimuli and biochemical reagents at the inner lumen and outside of the scaffold [16]. Among the mechanical stimuli, in particular, the intermittent shear flow by hydrostatic pressure and the shear stress by flow media were relevant to improving efficacy for differentiation of the epithelial and muscle lineage compared to steady shear flow.

Similarly, Eun-Jae Chung et al. utilized both supporting rod bioprinting and electrospinning and developed an esophageal scaffold reinforced by a 3D-printed PCL ring [14]. After bioprinting the reinforcing ring on the rod, a thin PCL layer was formed by electrospinning to form a nano-structured tubular structure. The printed tubular structures were wrapped into the omentum of rats for 2 weeks and then orthotopically transplanted for a circumferential esophageal defect. When macroscopically observed in the in vivo study, no fistulas, necrosis of the anastomosis, or abscess formation was found in the surrounding of the operating sections.

Maohua Lin et al. used direct bioprinting to a fabricated esophageal tubular stent with spiral patterns that applied the optimized design by computational simulation [130]. The printed esophageal tubular stent consisted of a mixed biodegradable polymer of medical-grade thermal polyurethane (TPU) and PLA in optimum proportion to achieve appropriate mechanical stiffness and flexibility. The group of the tubular stent with 10% PLA was investigated as a remarkable condition in the anti-migration force, self-expansion force, and human esophagus epithelial cell viability.

### 5.2. Blood Vessel

Blood vessels are components of the circulatory systems with hollow tube structures in the tissue and organs [131]. These transport blood cells, oxygen, and nutrients throughout the body and receive the CO_2_ and waste from the metabolic activity of the peripheral cells and tissues. Blood vessels are divided into arteries, veins, and capillaries according to their structural characteristic and biological functions. In general, the artery and vein walls consist of three layers: tunica intima (squamous endothelium), tunica media (smooth muscle cells), and tunica adventitia (fibrous collagen) [132,133].

Generally, blood vessel disorder refers to the hardening, enlargement, and narrowing of arteries and veins. These health problems trigger arterial diseases, which can cause death, such as coronary artery heart disease, cardiovascular disease, peripheral artery disease. Worldwide annual mortalities related to cardiovascular disease are expected to rise to 23.3 million by 2030 [134]. To date, revascularization strategies have included the stent, surgical bypass grafting, and angioplasty. Also, commercialized off-the-shelf alternatives, such as polytetrafluoroethylene (PTFE), gore-tex, and dacron, are also being proved clinically effective when replacing large-diameter vessels (≥6 mm). When using a small-diameter (≤6 mm) vascular graft, however, it has caused a thrombosis event with the closing lumen and the lack of long-term patency as well as intimal hyperplasia. Considering the limitations of current vascular grafts, tissue-engineered vascular graft (TEVG) has been developed using 3D bioprinting technology. In particular, for the clinical applications of the TEVG, anti-thrombosis and long-term patency overcome the essential issues. Recently, to achieve this goal, several interesting studies have reported generating a tubular structure with a biochemical component capable of physiological remodeling (Table 3).

**Figure 4:**
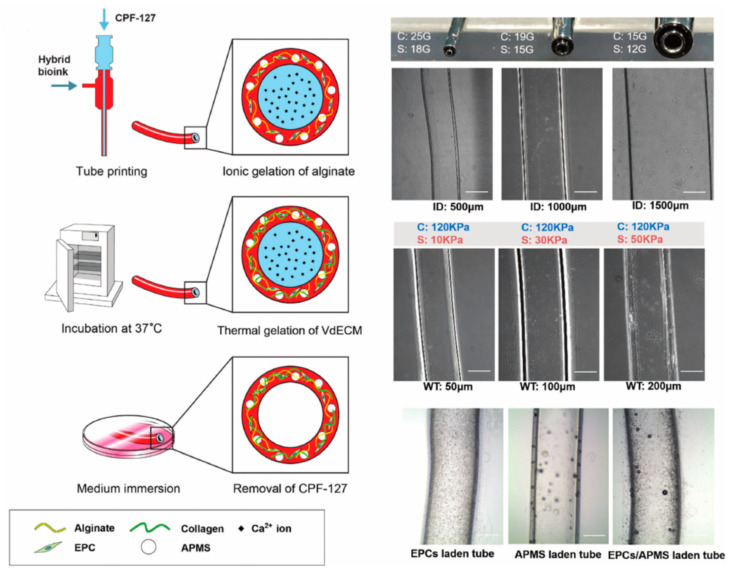
Schematic illustration and structural images of the vascular tubular structure by co-axial bioprinting [59].

Gao et al. successfully fabricated the tubular bio-blood-vessel (BBV) with hybrid bioink (a mixture of VdECM and alginate) using the versatile 3D co-axial bioprinting method (Figure 4) [59]. The VdECM/alginate hybrid bioink containing the atorvastatin-loaded PLGA microspheres (APMS) and endothelial progenitor cells (EPCs) provides a favorable environment to promote the proliferation and neovascularization. When bioink encapsulating APMS/EPCs is used with a core–shell nozzle, the inner shell is filled with CaCl_2_ solution (CPF127) for ionically crosslinking by releasing the calcium ion. The co-axial cell-printed tubular structure has been estimated in an ischemia model in nude mouse hind limb. It has induced an increased rate of neovascularization and the remarkable regeneration of ischemic limbs. The noteworthy point is that this research has achieved the creation of tubular structures with a broad range of diameters by controlling the core–shell nozzle. In addition, functional encapsulation of the cell/drug-laden bioink has shown potential for expansion as the printed BBV with the carrier that enables anti-thrombosis and long-term patency.

Sebastian Freeman et al. developed fibrin-based vascular constructs using rod supporting bioprinting [135]. The printable bioink consists of the fibrinogen with gelatin to achieve the desired shear-thinning property for self-standing. Unprintable fibrinogen was used as a printable biomaterial by blending the favorable rheological properties with heat-treated gelatin. During two months of the cultures after bioprinting, the burst pressure of the tubular structure reached 1110 mm Hg, and the remarkable improvement of the tensile mechanical properties was achieved in both the circumferential and axial elastic moduli.

Sang Jin Lee et al. combined rod supporting bioprinting and electrospinning for mechanical robustness and to build multi-layered structures using synthetic polymers (e.g., PCL) [136]. To induce potent angiogenic activity, the printed tubular structure was coated with polydopamine (PDA) and vascular endothelial growth factor (VEGF) on the surface. The coated-PDA layer enhanced the ability of the hydrophilicity; it also remarkably increased the vascular cell proliferation and angiogenic differentiation during in vivo/in vitro.

Quing Gao et al. fabricated hydrogel-based perfusable vascular structure with multilevel fluidic channels in tubular tissue approaches by using the 3D bioprinting that combined with co-axial and rod supporting bioprinting [31]. Partially cross-linked hollow alginate containing the fibroblasts and smooth muscle cells were extruded through a two co-axial nozzle and then printed along with a rotating supporting rod. The printed tubular structure exhibited a sufficiently strong mechanical strength (ultimate strength: 0.148 MPa) for the implantation due to the fusion of adjacent crosslinking reaction. Encapsulation of the fibroblast in the tubular structure showed over 90% survival within 1 week in vivo. This research has shown the ability to directly fabricating a perfusable vessel-like structure by cell-laden biomaterials through a coupled co-axial bioprinting and rod supporting method.

Thomas J. Hinton et al. 3D bioprinted a more complex structure than the perfusable arterial tree with alginate bioink and embedded it in the gelatin slurry support bath using a support bath-based bioprinting technology [120]. Perfusion structures mimicking a portion of the right arterial tree obtained through MRI data were printed in multiple branches with 3D tortuosity. Houman Savoji et al., similarly, printed vascular tubes (using core–shell nozzle) via freeform reversible embedding of photocrosslinkable bioelastomer prepolymers within a carbomer hydrogel bath by co-axial bioprinting and support bath-based bioprinting [137]. This tubular tissue-engineered approach to create a further advanced tubular structure made the significant achievement of mechanical robustness and recreated complex 3D anatomical architectures.

Kolesky et al. fabricated embedded vasculature constructs, repleted with multiple types of cells and an extracellular matrix (ECM), using direct bioprinting [138]. An aqueous fugitive ink composed of pluronic 127 was used for easy printing and removing under mild conditions to create vascular channels. In addition, gelatin methacrylate (GelMA) was used as a bulk matrix and cell carrier. After infilling and photopolymerizing the GelMA matrix on the fugitive pluronic 127 ink, the fugitive ink was removed by cooling the printed constructs below 4 °C, yielding open channels to fabricate the embedded vasculature constructs. Using this 3D bioprinting process, the potential of 3D vascular embedded constructs with human neonatal dermal fibroblasts (HNDFs) and human umbilical vein endothelial cells (HUVECs) was convincingly demonstrated. This result showed the development possibility for remodeling heterogeneous tissue constructs containing vasculature and multiple cell types.

### 5.3. Trachea

The trachea is a tubular structure in which the lower respiratory tract begins and refers to the pathway that begins immediately running between the larynx and the bronchi. The trachea is a composite tubular structure consisting of epithelium, basement membrane, connective tissue, smooth muscle, and cartilaginous layer. The tubular shape is about 2 cm in diameter and 11 cm in length with a flat posterior. The trachea acts as an airway to enter and exit the air during respiration. In addition, when debris, such as dust, enters into the trachea with air, it functions to move and remove the debris using ciliary movement and mucus [139,140].

Tracheas are becoming increasingly damaged due to severe environmental pollution. In addition, damage to the trachea has become a serious problem due to the increased use of ventilators for the treatment of patients [141]. In order to solve this problem, the transplantation of donor tissue from a deceased person to an injured organ has been reported. However, not only is it difficult to obtain donor tissue but even if it is obtained, there is a disadvantage that it can be transplanted through a complex pretreatment process over a long period of time [142]. The method of treating organ damage depends on the extent and length of the involvement of the site of injury. End-to-end anastomosis is a common treatment for circumferential injuries. However, end-to-end anastomosis has disadvantages, such as continuous endotracheal intubation, rupture, or stenosis of anastomosis after surgery. In addition, end-to-end anastomosis cannot be applied if more than 50% of the trachea needs to be excised. Such cases are difficult to treat clinically [143,144]. Tissue engineering is an appropriate approach to solve these problems; in addition, recent advances in 3D bioprinting technology have enabled the production of more sophisticated and systematic artificial structures (Table 4) [145,146].

Daisuke Taniguchi et al. developed an artificial trachea using kenzan method bioprinting [147]. They assessed the circumferential tracheal replacement using scaffold-free trachea-like grafts generated from spheroids consisting of several types of cells—chondrocytes, endothelial cells, and mesenchymal stem cells—to build 3D structures. This artificial trachea from spheroids was matured in a bioreactor and transplanted into a rat. In the transplantation, they used silicone stents to prevent collapse. As a result, chondrogenesis and vasculogenesis could be observed in this artificial trachea.

Manchen Gao et al. printed a biodegradable reticular PCL scaffold with similar morphology to the rabbits’ native trachea by direct bioprinting [148]. Chondrocytes were cultured in this 3D scaffold and conducted into the subcutaneous of nude mice. The scaffold showed the successful reconstruction and the proper supporting force to maintain the lumen as well as presenting remarkable cartilaginous properties both in vitro and in vivo.

Cheng-Tien Hsieh et al. fabricated a tissue-engineered trachea with structural similarity to the native trachea from water-based biocomposite ink at low temperature using direct bioprinting [149].

In that research, two kinds of water-based biodegradable polyurethanes with different physicochemical properties were used as biocomposite ink. The human MSCs were seeded into this tracheal construct, and then the construct was implanted in nude mice. After 6 weeks, the results showed dynamic compression moduli of the scaffolds that were 0.3–0.8 MPa under the force of 0.1–0.8 N, which was similar to the native trachea. It also confirmed gas-tightness by airflow test at positive and negative air pressures. Moreover, MSCs seeded in the tracheal scaffolds were grown into cartilage-like tissue. It expressed chondrogenic potential and secreted glycosaminoglycans (GAGs) and collagen after 14 days in vitro culture without any exogenous growth factors, such as bioactive factors or small molecular drugs. They showed that the tracheal scaffold of biocomposite inks and 3D bioprinting techniques might be used to fabricate personalized artificial tracheas for clinical applications. It also showed the possibility of incorporating exogenous growth factors in the water-based biocomposite ink to enhance the chondrogenesis of the MSCs.

**Figure 5:**
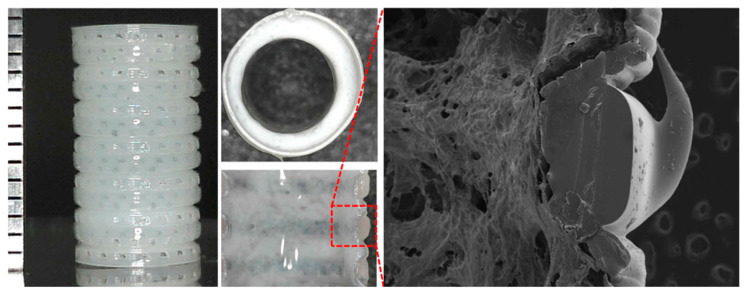
Structural images of the tracheal tubular structure by direct and rod-supporting bioprinting [150].

Jae Yeon Lee et al. developed an artificial tracheal structure PCL framework by extrusion bioprinting and silicone band by direct bioprinting and rod-supporting bioprinting (Figure 5) [150]. In particular, the states of the PCL extrusion were precisely controlled to create dotted circular patterns so that the bellows framework had about 300 µm pores in the wall except for groove parts. Then, they used a rod supporting bioprinting to print ring-shaped bands into the outer grooves of the PCL framework using a medical-grade silicone elastomer. The PCL framework was put around the rotating rods and rotated. They proved the potential of this artificial scaffold to be applied immediately in emergencies.

## 6. Future Perspectives and Concluding Remark

The biofabricated tissue-engineered tubular constructs require particular features of target-specific mechanical properties, anatomical accuracy, autoimmune acceptance, long-term patency, and similar cell arrangement for creating and mimicking of native tissues. This represents a significant technical challenge, and, to date, clinically meaningful tubular structure bioprinting approaches have been reported, utilizing versatile additive manufacturing techniques and biocomposite inks.

Various 3D bioprinting methodologies have emerged in the field of tissue engineering, and advanced technologies have been secured through rapid technological developments. Among them, the extrusion-based bioprinting system has been actively used to fabricate tubular structures with advantages of ease construction and flexibility in the use of various biomaterials. The fabrication of multi-layered tubular structures using supporting rods has been actively used, and approaches with more histologically close multi-cellular components using cell-spheroids have been developed. Co-axial bioprinting has also established itself as a promising approach that allows easy fabrication of freely adjustable perfusable tubular structures. In addition, this technology can be loaded with a variety of functional drugs, as well as cells that help bioenvironment cues, which are used in expandability platforms, such as vascular-embedded organoids and drug-screening devices. Support bath-based bioprinting has the advantage of being able to produce free- and multi-branched self-supporting forms using hydrate materials.

Despite the breakthrough in 3D bioprinting technology, artificial tubular structures of the esophagus, blood vessels, and trachea still face challenges for application as clinical substitutes [151,152,153]. This is because these tissues are exposed to high clinical needs, such as contraction, expansion by peristalsis, and blood pressure. In particular, the esophagus and trachea inevitably contact with external contaminants, such as liquid, food, and air, after insertion of the artificial tubular structure, thus hindering the growth of the functional endothelial layer. In addition, in the case of blood vessels, the absence of the endothelium layer may induce thrombus and stenosis. Therefore, pre-maturation, such as omentum culture, of bioreactor is considered a significant factor in the growth of artificial tissues before surgical approaches. In addition, many researchers are working to rebuild tubular organs, such as the stomach, intestine [154], bladder [9], and urethra [122], as well as the mentioned ones in this review. Thus, future developments of artificial tubular tissue should simultaneously entail the promising benefits provided by 3D bioprinting as well as the development of functional biocomposite inks and optimal cell culture techniques for target-tissues.

## Figures and Tables

**Table 1:** Advantages and disadvantages of the extrusion-based 3D bioprinting.

Extrusion-Based 3D Bioprinting	Advantages	Disadvantages	Reference
Co-axial bioprinting	- The building of a hollow construct with functional biological components - Fabricating permeable vascular-embedded 3D constructs - Manufacturing small-diameter vascular structures with endothelial and smooth muscle layers - Printing long-length warping vascular structures in a short amount of time	- Difficulty in making anatomical bifurcate structures - Difficulty in stacking hierarchical constructs	[59,88,114]
Kenzan method bioprinting	- High cell density - Reduced printing time on spheroid bioprinting	- The requirement of the additional cell spheroid fabrication process - Fixed interneedle distance	[115,116,117,118]
Rod supporting bioprinting	- Manufacturing ability of the self-supporting multi-layer hollow structure with target-specific cell components - Sequential printing using two or more printable biomaterials	- Dependence on rotating rod shape	[31,119]
Support bath-based bioprinting	- Enabling 3D bioprinting of the hydrate biomaterials - Creating a complex 3D anatomical architecture	- Limitation of available biomaterials - High cost	[120,121]
Direct bioprinting	- High freedom of shape	- Long production time	[122,123]

**Table 2:** The 3D bioprinting technique and biocomposite ink for the esophageal tubular structure.

3D Bioprinting Technique	Biocomposite Ink	Reference
Kenzan method bioprinting	Cell spheroids with human dermal fibroblasts, human esophageal smooth muscle cells, human bone marrow-derived mesenchymal stem cells, human umbilical vein endothelial cells	[115]
Rod supporting bioprinting and electrospinning	Polyurethane (PU), polycaprolactone (PCL)	[15]
Rod supporting bioprinting and electrospinning	Polycaprolactone (PCL)	[14]
Direct bioprinting	Thermal polyurethane (TPU), polylactic acid (PLA)	[130]

**Table 3:** The 3D bioprinting technique and biocomposite ink for the vascular tubular structure.

3D Bioprinting Technique	Biocomposite Ink	Reference
Co-axial bioprinting	Vascular-tissue-derived decellularized extracellular matrix (VdECM) with alginate	[59]
Rod supporting bioprinting	Fibrinogen and gelatin	[135]
Rod supporting bioprinting and electrospinning	Polycaprolactone (PCL)	[136]
Co-axial bioprinting and rod supporting bioprinting	Alginate	[31]
Support bath-based bioprinting	Alginate and gelatin slurry support bath	[120]
Co-axial printing and support bath-based bioprinting	Photocrosslinkable bioelastomer prepolymers ink (dimethyl itaconate. 1,8-ictanediol and triethyl citrate) and carbomer gel bath	[137]
Direct bioprinting	Pluronic 127 and gelatin methacrylate (GelMA)	[138]

**Table 4:** The 3D bioprinting techniques and biocomposite inks for the tracheal tubular structure.

3D Bioprinting Technique	Biocomposite Ink	Reference
Kenzan method bioprinting	Cell spheroids with chondrocytes, endothelial cells, and mesenchymal stem cells	[147]
Direct bioprinting and rod-supporting bioprinting	Polycaprolactone (PCL), silicone	[148]
Direct bioprinting	Polyurethane (PU)	[149]
Direct bioprinting	Polycaprolactone (PCL)	[150]
